# The experience of acculturative stress-related growth from immigrants’ perspectives

**DOI:** 10.3402/qhw.v8i0.21355

**Published:** 2013-09-25

**Authors:** Junhyoung Kim, Hakjun Kim

**Affiliations:** 1 Department of Recreation, Parks, and Leisure Services Administration, Central Michigan University, Mt. Pleasant, MI, USA; 2 Department of Tourism and Leisure Management, Kyung Hee Cyber University, Seoul, South Korea

**Keywords:** Acculturative stress, stress-related growth, older Korean immigrants, meaningful activity

## Abstract

Previous literature has mainly focused on the positive effects of stress associated with disability and illness, called stress-related growth. Little research has explored positive changes as a result of acculturative stress among a group of immigrants. In particular, older Asian immigrants may experience a high level of stress related to acculturation because they may face more challenges to adapt to and navigate a new culture. This study was designed to capture the characteristics of stress-related growth associated with acculturative stress. Using in-depth interviews among 13 older Korean immigrants, three main themes associated with the stress-coping strategies were identified: (a) the development of mental toughness, (b) engagement in meaningful activities, and (c) promotion of cultural understanding. These themes indicate that by following the stressful acculturation process, participants developed a better understanding of the new culture, engaged in various leisure activities, and enhanced mental strength. This finding provides information on how immigrants deal with acculturative stress and have positive psychological changes, which results in a sense of happiness and psychological well-being.

During the process of adapting to and/or navigating a new culture and society, immigrants may encounter numerous challenges, such as language barriers, cultural and ethnic differences, transportation difficulties, and cultural conflicts. Much research suggests that the acculturation process entails a certain level of stress that occurs because of a cultural gap between the ethnic and the new host culture (e.g., Berry, 1997; Lueck & Wilson, 2010). If the line of a cultural gap is expanded, the level of stress may be increased, which negatively affects immigrants’ health such as causing negative reactions to cultural differences and tensions and negative psychological symptoms (e.g., depression and anxiety) (Smart & Smart, 1995; Sodowsky & Lai, 1997; Thomas & Choi, 2006).

In particular, older Asian immigrants may experience a high level of stress related to acculturation because they may face more challenges to adapt to and navigate a new culture. Substantial evidence suggests that older East Asian immigrants experience more adaptation difficulties and perceive more acculturative stress than younger immigrants (Berry, 1992; Diwan, Jonnalagadda, & Balaswamy, 2004; Oh & Sales, 2002). The primary reason for this evidence is that older East Asian immigrants have a limited proficiency of English and communication problems with others (Lai, 2004; Nicassio, 1983; Weisman et al., 2005). These studies show that limited English skills resulted in social isolation and negative psychological symptoms, such as depression and anxiety, among older East Asian immigrants. In addition, it would be challenging for older East Asian immigrants to modify and embrace new cultural values and beliefs because they have a tendency to maintain their own life patterns, traditions, customs, values, and beliefs (Weisman et al., 2005). Based on these factors, a variety of researchers have identified older East Asian immigrants’ stressors such as cultural and racial conflicts, communication issues, their dependency on their children related to transportation and financial support, and racial discrimination experiences (e.g., Hwang, Wood, & Fujimoto, 2010; Mui & Kang, 2006).

In spite of adaptation challenges and acculturative stress, a growing body of literature suggests that stressful life events can lead to positive changes (e.g., Calhoun & Tedeschi, 2006; Tedeschi, Park, & Calhoun, 1998). These positive life changes associated with stress are labeled as *stress-related growth* (Park, 2006). According to Tedeschi and Calhoun (2004), as a result of traumatic and stressful life experiences, individuals are able to develop personal strength, pursue new possibilities of life, strengthen meaningful relationships, gain an appreciation of life, and enhance spiritual growth. Empirical evidence shows that individuals experience positive psychological changes from various types of disabilities and illnesses, such as HIV/AIDS (Cadell, Regehr, & Hemsworth, 2003), negative daily stressors (Losavio et al., 2011); cancer (Antoni et al., 2001; Garland, Carlson, Cook, Lansdell, & Speca, 2007), bereavement (Calhoun, Tedeschi, Cann, & Hanks, 2010; Michael, Dale, Rebecca, & Brian, 2008), and domestic violence (Cobb, Tedeschi, Calhoun, & Cann, 2006). These studies indicate that individuals who have encountered various stressful circumstances grow and thrive through stress-related growth.

Acculturative stress may be considered as an extended line of daily stressors for older East Asian immigrants because they experience various adaptation challenges and stressful life events (e.g., language barriers, cultural and ethnic conflicts, and changes in family values). From an applied stress-related growth perspective, acculturative stress may serve as a factor that produces positive changes for older East Asian immigrants, such as creation of coping strategies and resources and psychological strength. For example, Kim, Suh, and Heo (2012) suggested that adolescent Korean immigrants experienced positive psychological changes as a result of stressful intergroup contact and acculturation, including psychological thriving, cultural awareness, and cross-group friendships. They suggested that stressful interracial interactions and adaptation difficulties enabled Korean immigrant adolescents to develop coping strategies and experience stress-related growth. In addition, Kim et al. (2012) explored coping strategies associated with acculturative stress among Korean immigrant adolescents. They found that Korean immigrant adolescents developed positive emotions and fostered social relationships with other groups as a result of acculturative stress.

Unfortunately, no previous studies have explored the experience of stress-related growth associated with acculturative stress among older immigrants. Therefore, the purpose of this study was to capture the characteristics of stress-related growth that occurs as a result of acculturative stress from the perspective of older immigrants.

For this study, we selected older Korean immigrants for two reasons. First, demographically, older Korean immigrants are one of the fastest growing ethnic groups in the United States and this ethnic group is an understudied one (Berkman & Ko, 2008). Another reason is that older Korean immigrants perceive more acculturative stress and encounter more adaptation difficulties than other older Asian immigrants (Jang & Chiriboga, 2010; Sung, 2001). Based on these reasons, this study provided insightful information for future research related to acculturation and stress-related growth.

## Related literature review

### Acculturative stress among older Asian immigrants

The process of navigating and adapting to a new culture unquestionably entails some levels of stress. Stress caused by the acculturation process has been labeled as “acculturative stress” and Lueck and Wilson (2010, p. 48) defined it as “a reduction in mental health and well-being of ethnic minorities that occurs during the process of adaptation to a new culture”. Berry (1992, 1997) identified physical, social, cultural, and functional elements that cause acculturative stress. According to Berry (1992, 1997), physical stressors are related to a new social environment such as different social structures and systems; limited social supports, social networks and homesickness create social stressors; cultural stressors occur due to differences in culture, languages, customs, and traditions; and functional stressors are accompanied with financial situations, transportation, language barriers, and changes in family structures.

Substantial evidence shows that older East Asian immigrants experience various acculturative stressors including limited resources due to their dependency on their children, discrimination, cultural conflicts, language problems, and transportation problems (Hwang et al., 2010; Mui & Kang, 2006; Tran, 1990). Among various acculturative stressors, a lack of English skills is identified as the most difficult factor of adaptation among older Korean immigrants (Lee & Holm, 2011; Mui, 1996). Because of limited English skills, older East Asian immigrants may have difficulties in communicating with other ethnic groups and limited opportunities to embrace new cultures. Experiencing differences in culture, social norms, and values are associated with acculturative stress, which negatively influences older Asian immigrants’ health (Kim, 1999; Lai, 2004).

In addition, older East Asian immigrants tend to maintain their own cultural values and beliefs, which create conflicts with their adult children who are highly acculturated, and misunderstandings with others who are culturally or ethnically diverse (Weisman et al., 2005). For example, Mui and Kang (2006) found that the cultural gap between older Asian immigrants and their adult children caused depressive symptoms, which negatively affected their health. Lai (2004) also found that differences in cultural values and beliefs were the main predictors of depressive symptoms. Because of adaptation challenges such as the cultural gap and differences, acculturative stress has been negatively associated with a perception of health and psychological well-being among older Asian immigrants (Berry, 1980, 1997; Cheung-Blunden & Juang, 2008).

### Stress-related growth

A growing body of literature provides evidence that individuals experiencing major life events associated with trauma and stress have positive psychological changes (e.g., Park, Cohen, & Murch, 1996; Tedeschi & Calhoun, 2004). Following distressing and traumatic life experiences, individuals may engage in determined efforts to cope with stressors and develop their own coping strategies (e.g., Helgeson, Reynolds, & Tomich, 2006; Janoff-Bulman, 2004; Tedeschi & Calhoun, 2004). As a result, individuals are able to experience a wide range of positive outcomes, such as enhanced self-esteem, positive spiritual changes, social support, and coping resources (e.g., Linley & Joseph, 2004; Tedeschi & Calhoun, 1995, 2004). For example, women with HIV/AIDS experienced various stress-related growth benefits such as positive behavioral changes, spiritual growth, meaningful relationships, positive self-image, appreciation of life, and goal-oriented behaviors (Siegel & Schrimshaw, 2003).

According to Tedeschi and Calhoun (1996, Tedeschi and Calhoun 2004), following the aftermath of struggle with stressful events, individuals experience changes in personal strength, pursue new possibilities of their lives, gain an appreciation of life, improve meaningful relationships, and develop spiritual growth. Park and colleagues (1996) identified four major domains in which individuals facing stressful life circumstances grow and thrive: (a) broadened life perspectives, (b) new coping skills, (c) enhanced personal resources, and (d) deepened meaningful relationships. Stress-related growth has been associated with psychological health, such as optimism, positive affect and psychological and subjective well-being (e.g., Bostock, Sheikh, & Barton, 2009; Bower, Moskowitz, & Epel, 2009; Durkin & Joseph, 2009).

Some studies have examined what factors contribute to stress-related growth. Prati and Pietrantoni (2009) conducted a meta-analysis of 103 studies to identify factors contributing to stress-related growth. They found that various coping strategies (e.g., acceptance, reappraisal, religious, and support), optimism, and social support served as important elements in facilitating stress-related growth. In particular, positive reappraisal and religious coping strategies are more associated with stress-related growth than optimism and social support. Park and Fenster (2004) explored predictors of occurrence of stress-related growth and demonstrated that cognitive and coping processes (e.g., making meaning, resources, appraisals, and coping activities) predicted stress-related growth. This study indicates that individuals who make conscious and determined efforts to heal and utilize various coping resources are more likely to experience stress-related growth. In support of Park and Fenster's (2004) findings, Finzi-Dottan, Triwitz, and Golubchik (2011) found that emotional intelligence (a cognitive process) was the most predicting factor in facilitating stress-related growth among parents of children with attention deficit/hyperactivity disorder.

## Methods

To capture the characteristics of stress-related growth associated with acculturative stress among older Korean immigrants, we conducted semi-structured in-depth interviews. According to Zhang and Wildemuth (2009), in-depth interview enables researchers to garner detailed information about individuals’ life experiences, perceptions, feelings, emotions, and attitudes.

### Participants

A criterion-based sampling strategy suggested by Strauss and Corbin (1998) was used to describe and interpret experiences and mechanisms of stress-related growth among older Korean immigrants. The criteria for the participants were that they (a) had immigrated to the United States from Korea; (b) were 65 years old or older; and (c) had lived in the United States for at least 10 years, a number again based on previous research (i.e., Hurh & Kim, 1984; Kim, Kleiber, & Kropf, 2001).

Participants were recruited with the cooperation of Korean communities (e.g., Korean churches, Korean markets, and Korean senior centers) in the northwestern United States. Only the last name (a pseudonym) was used to identify participants. All of the participants voluntarily participated in this study. The university Institutional Review Board approved these procedures. No payment was offered for participation in this study.

A total of 13 participants participated in this study. Seven of the participants were male and six were female. The participants in this study ranged in age from 65 to 82 (M=68.8). Four were widows, and the average length of time since their immigration was 32 years (see Table I). The sample size was determined on the basis of theoretical saturation, as suggested by Mack, Woodsong, MacQueen, Guest, and Namey (2005). Guest, Bunce, and Johnson (2006) indicated that if researchers conduct qualitative work with a sample from a relatively homogeneous group (i.e., culture, race, ethnicity, or gender), 12 participants are generally sufficient to reach saturation. In this study, 13 participants appeared to be adequate as we reached a saturation point.

**Table I:** Demographic characteristics of participants

Name	Age (years)	Gender	Length of stay (years)	Educational background	Marital status
Lee	77	Female	10	Bachelor	Widow
Park	73	Male	33	High school	Married
Jo	70	Female	33	High school	Married
Kwon	70	Female	45	High school	Married
Lee-Si	70	Male	25	High school	Married
Chen	66	Female	25	High school	Married
Kim	65	Male	32	PhD	Married
Lee-J	80	Male	52	PhD	Married
Choi	74	Male	46	Bachelor	Widow
Lee-W	65	Male	26	High school	Married
Kwon-F	65	Female	34	Bachelor	Widow
Jang	68	Male	43	PhD	Married
Jeun	82	Female	15	High school	Widow

### Data collection

Each interview lasted between 50 and 90 min. The research team developed the interview questions to capture the characteristics of stress-related growth related to acculturation. As preferred by participants, all interviews were conducted in Korean and all participants permitted the researcher (the first author) to record the conversation. A grand and mini-tour question strategy suggested by Spradley (1979) was employed to obtain more data from participants. Examples of grand tour questions were “Do you feel most comfortable speaking in English or Korean?”, “When did you move to the United States?”, and “Could you tell me about your life story?” To capture positive changes related to acculturation, mini-tour questions were asked such as “Have you gained any benefits when you encountered adaptation challenges? If so, what were some of these benefits?”, “How did you deal with the challenges in your life during your immigration processes?”, and “Have you felt any positive changes in your life because of adaptation challenges? If so, please describe some of these changes.”

At the end of the interview, participants were asked to answer questions regarding their age, marital status, educational background, and length of stay in the United States. This information was used to profile participants.

### Data analysis

To analyse the data, we followed the five steps presented by Creswell (2009): (a) creation of raw data; (b) preparation of data analysis; (c) generation of general themes; (d) data coding; and (e) interpretation of themes. Based on these steps, the constant comparative data analysis was used to identify emerging themes and constantly compare one set of data to another (Merriam, 1998).

1. *Creation of raw data*. The first author produced each transcription in Korean, and the research team invited two research professionals to verify the translations from Korean to English. They were involved in the process of the preparation of the data and participated in the back-translation process, which increased the credibility of the data.
2. *Preparation of data analysis*. With the cooperation of two research professionals, all transcriptions were organized for analysis.
3. *Generation of brief ideas*. The research team read each transcription repeatedly to understand participants’ experiences and briefly generated the brief ideas that were similar and/or contrasting with each other.
4. *Data coding*. Based on the brief ideas, the research team created open coding categories such as adaptation challenges, positive and negative experiences of acculturation, and coping strategies. With open coding schemes, the researchers created the general themes and summarized them with direct quotes.
5. *Interpretation of themes*. The general themes with direct quotes allowed the research team to identify specific key themes that satisfied the purpose of this study. We interpreted and analysed the key themes and incorporated them with the meanings of the core themes. This process was conducted concurrently and the research team fully discussed themes to interpret and solidify the data.

### Trustworthiness in qualitative research

Among various methods of improving the credibility of the data, we employed member checking, expert review, and back-translation. According to Lincoln and Guba (1999), inviting participants to review the data analysis helps to increase the reliability of the data. The research team provided a summary of the themes to the participants to increase the credibility of data analysis. Previous qualitative researchers emphasized the idea that back-translation may be necessary to improve the quality of translation from one language to another, which enhances the credibility of the data (Guillemin, Bomardier, & Beaton, 1993; Suh, Kagan, & Strumpf, 2009). To employ back-translation, the research team invited two bilingual research professionals to participate in the back-translation process. They agreed that there were no problems with the conceptual meanings and contents of the translations. After the back-translation process, a professional editor reviewed the transcriptions for proofreading. In addition, the research team invited two qualitative research professionals to review the transcriptions and elaborate on data analysis as a way of expert review. We captured and interpreted themes and reached an agreement associated with the identified and interpreted themes of the data (Lincoln & Guba, 1999).

## Findings

Through the analysis of the data, several key themes were captured related to the positive and negative immigration experiences among participants. Concerning negative aspects of immigrant life, participants faced numerous challenges associated with acculturation, such as language barriers, family issues, cultural and ethnic differences, children's struggles, and racial conflicts. In spite of the challenging acculturation process, participants experienced positive social and psychological changes. In this study, we focused on the experience of stress-related growth that occurs because of adaptation challenges and identified three main themes: (a) the development of mental toughness, (b) engagement in meaningful activities, and (c) promotion of cultural understanding. These themes indicate that by following the stressful acculturation process, participants developed a better understanding of the new culture, engaged in various leisure activities, and enhanced mental strength.

### The development of mental toughness

The development of mental toughness is one of the most salient themes emerging from the data. Negative immigration experiences challenged all of the participants to learn new cultural values and beliefs, but at the same time most participants developed mental toughness because of adaptation challenges such as a lack of cultural understanding and unfamiliar social norms. Participants mentioned that they cultivated their strength and endurance to overcome many adaptation challenges. In addition, participants developed self-discipline and the inner strength and mental endurance necessary for dealing with acculturative stress.

All participants experienced racial tension and communication issues, which caused acculturative stress. The majority of participants believed that acquiring new linguistic skills would be the most challenging thing in their lives, because they did not receive any education in the United States. Due to limited language skills, they mentioned that they had racial discrimination experiences such as being ignored and belittled by others. In particular, they said that it was challenging to express their feelings, thoughts, and opinions to others because of language barriers. Even though three participants had a higher education, they also shared racial discrimination experiences in a different social context which was mainly in educational settings.

In spite of adaptation challenges, participants mentioned that negative life experiences caused by a lack of English skills made them feel much stronger psychologically. In addition, such experiences enabled them to develop a sense of perseverance because they had to deal with various acculturative stressors. According to them, they decided to actively deal with any challenges and experienced mental toughness. Some participants who experienced racial tension and conflicts experienced mental toughness. For example, Park (male, 73), who runs his business in a specific neighborhood, experienced racial tension and racial conflicts. Previously, his customers had taken advantage of his situation as an immigrant and made fun of his appearance.

Under various circumstances, he developed his own mental strength and toughness to deal with any tensions and conflicts. He said:

When I began my business, many neighbors vandalized my store and shouted, ‘go back to your country’ and ‘learn English.’ I was so scared of making the report and did not say a word to them…. I became much tougher mentally and yelled at them. I just expressed my feelings and thoughts directly to them because I had family to take care of. To survive, I should deal with that.

He made a comment that his customers became much friendlier once he exhibited a strong sense of mental toughness.

Jang (male, 68) had taught science-related classes at a historically black university. He shared his story as a teacher; some students and faculty members made fun of his strong accent and sometimes ignored his instructions because of his ethnic background. He said, “at that time I was the only Asian on the campus” and thoughts that people around him stared at him like the way they would at the zoo. To overcome this circumstance, he invested his time and effort to be a good researcher and a scholar. He made significant academic accomplishments and developed a sense of perseverance. He said that because of experiencing racial discrimination he was able to demonstrate his teaching and research abilities and develop a sense of a strong mentality. As a result, faculty members and students expressed more positive attitudes toward him.

Ten participants experienced economic hardships when they immigrated to the United States. Because of various reasons (e.g., language barriers and lack of education), they had to get a job, whereas Americans did not prefer to work and worked diligently to provide necessities for their children. According to them, their top priorities were their children's education and happiness and they were happy to have an opportunity to sacrifice their lives for them. To provide any support for their children, they mentioned that they had to develop a sense of perseverance and mental strength. Some of them said that they should be mentally strong because they did not want to see their children become mentally weak because of them. For example, Lee-W (male, 65) immigrated to the United States because of his children's education and for the pursuit of a better life. To achieve his goals, he thought that he should be mentally ready to live in a totally different world. He said:

My wife and I had worked at least 15 hours a day and did not spend any time with my children. Even though we were physically exhausted and tired after work, we did not express it to our children because it might negatively affect their studies…. We worked so hard for our children and gradually became much stronger.

He also pointed out that diligence is one of the important cultural values in Korean culture and with such hard work he was able to raise his children successfully and develop a strong mentality.

Even though participants experienced numerous challenges associated with acculturation, they coped well with adaptation challenges and developed a sense of perseverance, which resulted in mental toughness and mental strength. In addition, participants used mental strength as a survival strategy for themselves as well as their children.

### Engagement in meaningful activities

The process of adapting to a new culture and society created the opportunities for participants to discover, explore, and engage in meaningful activities. According to all 13 participants, they were given an opportunity to become involved with various meaningful activities, such as church-based activities, volunteer activities, and culturally meaningful activities. They used engagement in meaningful activities as a way of coping with acculturative stress, which contributed to acculturation. For example, Jang (male, 68) mentioned that participating in Korean traditional games helped them to deal with acculturative stress and enhance their happiness.

In addition, participants believed that a new cultural environment facilitated their leisure motivations to engage in meaningful activities. For example, six participants were involved in volunteering activities. In many cases, they utilized their immigration experiences and resources to help other Korean immigrants who recently immigrated adapt to a new culture and society. They mentioned that it was common for recently immigrated Korean immigrants to face numerous challenges which were similar to those they had experienced before. To help other Korean immigrants, participants utilized their skills, knowledge, and information related to acculturation. According to Kwon (female, 70), she provided transportation and meals for other older Korean immigrants who could not drive. She said:

Helping new immigrants who were emigrating from Korea was my passion and love, even though we, too, were struggling to adapt to new societies. If newcomers needed a ride, we offered a ride and helped them settle in a new place. For 29 years, I have been providing a ride for one person.

She also provided meals for and supported other Korean immigrants who had encountered adaptation and psychological difficulties. She mentioned that she was willing to help recently immigrated Korean immigrants because she herself had faced numerous challenges associated with acculturation. By engaging in a volunteering activity, she was satisfied by the fact that she was able to provide emotional and social support for other immigrants. As a reward, she considered this volunteer activity to be a fulfilling, fun activity.

Similarly, Chen (female, 66) organized a social support group called “Enoch” for older Korean immigrants because they expressed negative feelings such as depression, loneliness, and isolation because of family conflicts and adaptation difficulties. She created a unique social support system which helped older Korean immigrants enhance a sense of belonging and connectedness with each other. As a result of her involvement in a volunteering activity, older Korean immigrants were connected with each other and expanded social networks. She also mentioned that she understood what the challenges of immigration life could be and started involving herself in various volunteer activities to help others who had difficulties.

The majority of participants were engaged in a variety of church-based events and activities. Through participating in these activities, they developed more faith and used spiritual peace and comfort to deal with adaptation challenges such as communication issues, racial conflicts, and children's education. There were a variety of church-based events in which participants were encouraged to participate such as Bible studies, missionary work, and volunteer work so that they developed feelings of emotional and social connectedness and developed their spiritual lives. Through these activities, they reported that they developed the positive aspects of their lives and relieved negative feelings, such as depression and loneliness. Such positive outcomes encouraged them to pursue church-based activities with enthusiasm and passion. For example, Lee-Si (male, 70) realized the value of spirituality in sharing life stories with each other through the Bible study and actively engaging in this activity. He also mentioned that as a result of their participation in this activity, many participants in the group had improved their negative feelings and emotions.

Five participants created and developed culturally meaningful activities as a way of delivering Korean culture and etiquette to others who were not familiar with them, such as Korean children who were born in the United Sates. In addition, culturally meaningful activities were developed and provided to make social connections for people who had connections to Korea, such as US veterans of the Korean War and people who had been adopted from Korea. For young adults and children who are not accustomed to Korean culture, participants that helped deliver knowledge and information about how to appropriately interact with older adults offered Korean language classes and culture-related events. They came up with various culturally meaningful activities because they realized the value of them as a way of delivering Korean culture and knowledge to others. For example, Choi (male, 74) became involved in culturally meaningful activities to develop an intimate relationship with US veterans of the Korean War and to make a relation with people who were adopted from Korea. He also noted that the adopted children were eager to gain some knowledge and information about Korea, such as the language, culture, and customs. As part of the group that shared common interests, they participated together in several traditional Korean games.

A new cultural environment enabled participants to create and develop various meaningful activities to provide social and emotional support for each other. As a result, they developed a sense of belonging, connectedness, and reduced negative life challenges.

### Promotion of cultural understanding

Promotion of cultural understanding is another theme that emerged from the data. While participants encountered cultural conflicts and misunderstandings with others because of cultural and ethnic differences, they acknowledged cultural similarities and differences and gained knowledge of how to interact with others in culturally appropriate manners. Such a positive process resulted in an enhanced sense of cultural understanding. Participants mentioned that they were exposed to learn different cultural values and beliefs and determined to accept differences in lifestyles. For example, Chen (female, 65) said that hastiness was one of the most widely known Korean cultural traits and preferred that everything be done fast. In particular, when she visited a hospital, she was frustrated with the long process of finding out test results and making appointments. During acculturation, she became more patient and took things slowly, which indicated that she enhanced her sense of cultural understanding.

In terms of cultural understanding associated with cultural conflicts, Jo (female, 70) said:

Regardless of how old I was, most Americans called me by my first name and based on my cultural value, this was unacceptable and considered rude. In Korea, there is a strict hierarchy related to age and people should use honorific languages with older adults. I struggled so much with the way others treated me because I regarded them as other Koreans. It was my fault because I realized I lived in a different country and they (Americans) had a different culture. Even though it took me a while to understand this difference, I started respecting their ways and now I am fine with that.

When Jo found any differences in culture, she sought to understand cultural and ethnic differences and developed an interpersonal relationship with others.

Four participants mentioned that they gained a sense of cultural knowledge through participating in activities, which helped them understand cultural differences. By playing golf, Kim (male, 65) made many American friends, which enabled him to learn English and better understand US culture. Similarly, Choi (male, 74) discovered cultural differences through his Judo activities:

I frequently attended some conferences and events related to Judo in the U.S. and Korea. In Korea, if different ideas and suggestions came up in these meetings, the people who had different ideas were more likely to dislike each other or even be enemies. So it was difficult to go against others’ opinions because I did not want to be seen as an enemy. In our culture, age is a very important factor that decides something important, so I respect opinions that come from others who are older than I am. However, in the U.S., even though they had different opinions and ideas, after the meeting, they smiled and became good friends. They shared many good ideas freely and I felt no restrictions related to age. This was a difference.

For Choi (male, 74), by interacting with Judo students, he gained cultural knowledge and mentioned that Judo activity provided him with an opportunity to understand different societies.

Six participants lived with their adult children and experienced cultural exchanges and cultural dynamics in a family setting. Through cultural exchanges with their adult children and grandchildren, participants mentioned that teaching and delivering Korean culture was challenging and stressful because they had a lack of understanding of their grandchildren's cultural identities. During acculturation, they realized the fact that their grandchildren who were born in the United States should maintain and develop US cultures even though they were incongruent with theirs. For example, Lee (female, 77) said that she realized that racially her grandchildren look like Koreans, but culturally they were not. She expressed a better understanding of where her grandchildren were from and accepted their cultural identities. With this better cultural understanding, Lee expanded her cultural understanding to other children who were born in the United States. She said:

At the church I thought that children lacked good manners and were rude because their behaviors and expressions had been undesirable based on my cultural values. Later, I realized that even though they look similar to me, they have different cultures and I just overcame my misunderstandings and accepted their identities.

In addition, according to Lee-Si (male, 70):

I teach Korean culture to my grandchildren. Sometimes, the third grandchild calls her older brother “John Park,” not “OPPA” (the Korean-specific honorific language used to address the older brother). I tried to understand this situation by reminding myself that English had become their first language, but I did my best to teach them how to call their brothers and sisters appropriately based on Korean etiquette…

While he exchanged cultural knowledge, Lee-Si promoted cultural understanding that using their first names would be accepted in his mind.

These examples exhibit that exposure to new cultures enabled participants to experience cultural conflicts and navigate new cultural values and beliefs. Through this acculturation process, participants were provided with an opportunity to have a better cultural understanding and gain cultural knowledge. In this way, promoting new cultures facilitates growth experiences among participants.

## Discussion

This qualitative research is an initial exploration of capturing stress-related growth that occurs as a result of acculturative stress among older Korean immigrants. Older Korean participants identified various adaptation challenges related to acculturation such as language barriers, cultural conflicts, limited social networks, children's education and cultural and ethnic differences, which resulted in acculturative stress. This research was designed to capture positive changes following acculturative stress and provided three salient themes identified as the characteristics of stress-related growth associated with acculturation: (a) the development of mental toughness, (b) engagement in meaningful activities, and (c) promotion of cultural understanding. These identified themes show that older Korean participants experienced stress-related growth that occurred because of adaptation difficulties.

Literature on stress-related growth suggests that stress and growth experiences coexist and individuals experiencing a major life event such as illnesses and/or disabilities grow and thrive as a result of trauma and stress (e.g., Park et al., 1996; Tedeschi & Calhoun, 1996). These studies demonstrate that stressful life events provided opportunities for individuals to put more value on life, appreciate their lives, find new possibilities of life, experience spiritual growth, and relate to others. The findings of this study support the idea that acculturative stress entails growth experience. Older Korean participants sought to cope with acculturative stressors and developed their own coping strategies, which resulted in stress-related growth. In addition, a new cultural environment provided opportunities for older Korean participants to develop a sense of perseverance and mental toughness, and it seems that such positive outcomes may serve as a coping strategy.

Previous empirical studies have mainly focused on positive changes and outcomes associated with trauma and stress among a wide range of individuals who had traumatic life experiences, such as cancer, HIV/AIDS, bereavement, combat, and domestic violence (e.g., Cadell et al., 2003; Michael et al., 2008). This study suggests that acculturative stress is an extended line of negative stressful life events that produce stress-related growth experience. Based on the findings of this study, the scope of a group of individuals experiencing stressful and traumatic life experiences has been expanded to a group of immigrants who gained positive outcomes because of acculturative stress.

In a recent study, Kim, Suh, and Heo (2012) captured stress-related growth experience during stressful intergroup contact and acculturation among Korean immigrant adolescents. The study by Kim et al. mentioned that stressful intergroup contact and adaptation difficulties enabled Korean immigrant adolescents to experience psychological thriving, enhance cultural and ethnic understandings, and foster cross-group friendships. The result of this study is consistent with Kim and colleague's finding that the characteristics of stress-related growth such as mental toughness, participation in meaningful activities, and cultural understandings were identified and reinforced by older Korean participants.

According to Prati and Pietrantoni's (2009) meta-analysis of stress-related growth, coping strategies (e.g., acceptance, reappraisal, religious, and support) and social support are the main contributing factors that contribute to stress-related growth. Based on the findings of this study, involvement in meaningful activities may be a contributing factor that contributes to stress-related growth. A few studies (e.g., Chun & Lee, 2008, 2010) support this finding that engagement in meaningful activities facilitated stress-related growth among individuals with spinal cord injuries. In a similar context, older Korean participants engaged in meaningful activities such as volunteering activities, church-based activities, and culturally meaningful activities and as a result, they provided emotional and social support for each other. In addition, older Korean participants used leisure as a way of dealing with various acculturative stressors, which contributed to positive outcomes such as happiness and a reduction in negative psychological symptoms. Therefore, this finding indicates that leisure may serve as an important vehicle for facilitating stress-related growth.

From a perspective of positive psychologists, any individuals have their own strengths, talents, abilities, and potential for positive outcomes such as hope, happiness, creativity, persistence, and tolerance (Seligman & Csikszentmihalyi, 2000). The idea of positive psychology is that such positive characters allow individuals to create their own coping strategies and coping resources to deal with life challenges. For example, Kim's (2012) study suggested that female Korean immigrants utilized their own strengths and personal assets as a way of reducing adaptation challenges and increasing social interactions with other ethnic groups. This study is aligned with the concept of positive psychology and supports Kim's study, as older Korean participants developed their own coping strategies and utilized coping resources to deal with acculturative stressors. As a result, they reported experiencing happiness, perseverance, and social support.

In summary, we attempted to create a concept of *acculturative stress-related growth*, which is defined as stress-related growth that occurs as a result of adaptation difficulties related to acculturative stress. Such a way of understanding the concept of acculturative stress-related growth may indicate that the acculturation process generates a certain level of stress for older Korean participants and also provides an opportunity for them to experience positive outcomes related to acculturative stress.

### Limitations and need for future research

There are some limitations that we should address. First, we attempted to capture the positive outcomes related to stress-related growth among older Korean immigrants. Members of this group may have different levels of acculturation and immigration experiences, which may influence their growth experience. In addition, participants’ educational backgrounds and gender may be additional factors that affect stress-related growth experiences. Future research is needed to investigate relationships among levels of acculturation, demographic backgrounds, and stress-related growth.

This study did not measure the actual level of participants’ acculturative stress. Depending on the level of acculturative stress, older Korean participants may experience different pathways of positive outcomes related to adaptation difficulties. It may be helpful for future researchers to examine the relationship between the level of acculturative stress and stress-related growth.

The last limitation is in regard to a limited population. In this study, older Korean immigrants were the participants and other immigrants from different countries may have different immigration experiences and acculturation processes. For example, western immigrants from England, New Zealand, and Australia may have different immigration experiences from immigrants from Asia because of the language matter. It may be interesting for future researchers to examine any cultural and ethnic differences and their effect on stress-related growth between western immigrants and eastern immigrants.

In spite of limitations, this study can expand the body of literature on stress-related growth related to acculturation. This also provides information on how immigrants deal with acculturative stress and have positive psychological changes. In particular, engagement in meaningful activities may serve as a catalyst for facilitating acculturation and coping with various acculturative stressors. Leisure professionals need to provide effective and valuable activities for immigrants so that they may enhance their own happiness and quality of life.
